# Identification of unique transcriptomic signatures through integrated multispecies comparative analysis and WGCNA in bovine oocyte development

**DOI:** 10.1186/s12864-023-09362-w

**Published:** 2023-05-18

**Authors:** Fa-Li Zhang, Wei-Dong Li, Geng Zhang, Min Zhang, Zhao-Jun Liu, Ke-Xin Zhu, Qing-Chun Liu, Shu-Er Zhang, Wei Shen, Xi-Feng Zhang

**Affiliations:** 1 College of Veterinary Medicine, Qingdao Agricultural University, Qingdao, 266100 China; 2 College of Life Sciences, Key Laboratory of Animal Reproduction and Biotechnology in Universities of Shandong, Qingdao Agricultural University, Qingdao, 266109 China; 3 College of Animal Science and Veterinary Medicine, Shandong Agricultural University, Tai’an, 271018 China; 4 Advanced Medical Research Institute, Shandong University, Jinan, Shandong China; 5 College of Animal Science and Technology, Nanjing Agricultural University, Nanjing, 210095 China; 6 Animal Husbandry General Station of Shandong Province, Jinan, 250010 China; 7 Qingdao Longming Cattle Industry Co., Ltd, Qingdao, China

**Keywords:** Cattle, WGCNA, Oocyte development, Comparative transcriptome, Transcriptomic signature

## Abstract

**Background:**

Cattle (*Bos taurus*) are a major large livestock, however, compared with other species, the transcriptional specificity of bovine oocyte development has not been emphasised.

**Results:**

To reveal the unique transcriptional signatures of bovine oocyte development, we used integrated multispecies comparative analysis and weighted gene co-expression network analysis (WGCNA) to perform bioinformatic analysis of the germinal follicle (GV) and second meiosis (MII) gene expression profile from cattle, sheep, pigs and mice. We found that the expression levels of most genes were down-regulated from GV to MII in all species. Next, the multispecies comparative analysis showed more genes involved in the regulation of cAMP signalling during bovine oocyte development. Moreover, the green module identified by WGCNA was closely related to bovine oocyte development. Finally, integrated multispecies comparative analysis and WGCNA picked up 61 bovine-specific signature genes that participate in metabolic regulation and steroid hormone biosynthesis.

**Conclusion:**

In a short, this study provides new insights into the regulation of cattle oocyte development from a cross-species comparison.

**Supplementary Information:**

The online version contains supplementary material available at 10.1186/s12864-023-09362-w.

## Background

The normal development of oocytes is critical for maintaining pregnancy, reproducing populations and maintaining genetic diversity [1,2]. However, the transcriptome signatures of bovine (*Bos taurus*) oocyte development, especially those unique compared to other species, have not been well characterised so far.

In female mammals, primordial germ cells undergo mitosis, meiosis and maturation to eventually develop into oocytes [3]. Interestingly, oocytes undergo a prolonged period of prophase arrest before puberty. Only after the preovulatory luteinizing hormone (LH) surge do, the germinal follicle (GV) oocytes initiate resumption of meiosis, expel the first polar body, and then reach metaphase of the second meiosis (MII), ready for fertilisation [4]. High levels of cyclic AMP (cAMP) in oocytes are thought to be critical for maintaining their long-term meiotic arrest [5]. Mechanistically, high levels of cAMP activate protein kinase A (PKA) and inhibit the expression of the meiosis-promoting factor (MPF) [6]. With development, the level of cAMP gradually decreases, leading to the reactivation of MPF, then oocyte meiotic resumption is morphologically characterised by the dissolution of the nuclear membrane, which is termed ‘germinal vesicle breakdown’ (GVBD) [7]. The characteristics of ovum development from GV to MII in sheep, pigs, and mice have been reported [8, 9], however, the specificity of oocyte development in cattle during this period remains an unanswered question.

It is reported that long non-coding RNAs (lncRNAs) participate in regulating the development of bovine oocytes from GV to MII. LncRNA have been reported to affect the development of vitrification at the GV stage in cattle [10]. Moreover, cAMP signalling [11], MPF, PI3K/Akt signalling [12] and fatty acid metabolism [13] are involved in regulation of accelerated maturation following meiotic arrest in cattle. This complex molecular network is considered to be important for the oocyte to resume meiosis, which in turn produces a fertilized oocyte.

RNA sequencing (RNA-seq) has been widely used to detect transcriptional information signatures of tissue samples, and differential expression analysis provides an important reference for identifying tissues or tissue development under different states [14]. To cope with the increasing amount of RNA-seq data, a large number of data processing workflow have been developed. A growing body of evidence suggests that WGCNA is not only helpful to construct a gene regulatory network, but also to identify hub genes in the network, which is beneficial for evaluating single or complex traits [15]. Overall, in this study, we exhaustively compared the transcriptional characteristics of bovine, sheep, porcine and mouse oocytes from the GV phase to MII phase, and identified 2371 bovine-specific regulatory genes. We found 173 genes related to bovine hub genes specifically related to oocyte development via WGCNA Further, we compared bovine-specific regulatory genes and hub genes, and obtained 61 unique bovine transcriptomic signatures.

## Results

### Transcriptional signatures of GV and MII phases in oocytes of bovine and other species

**Fig. 1:**
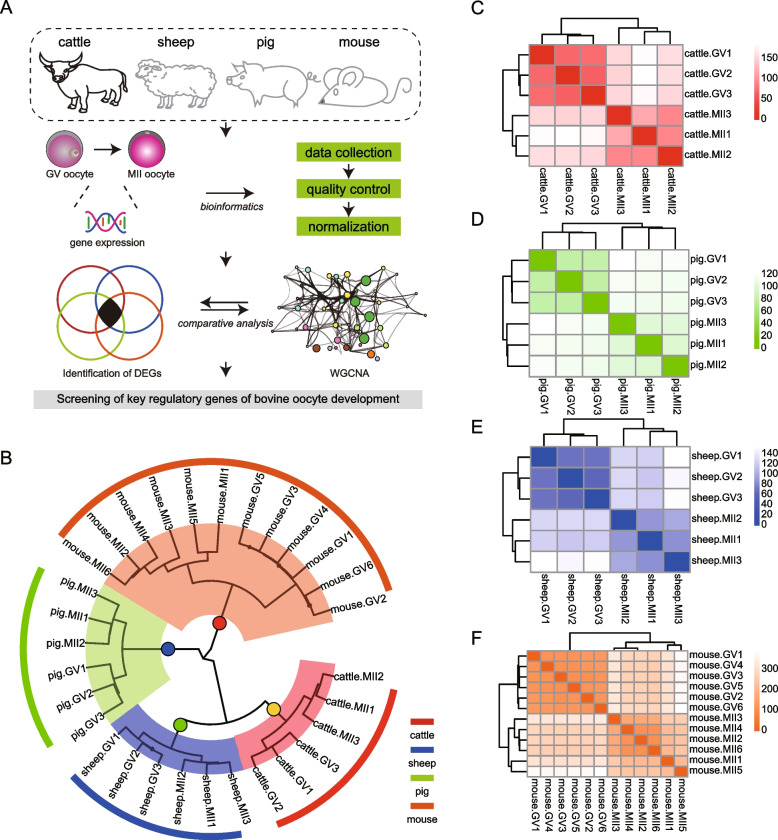
Transcriptome landscape of multispecies germinal vesicle (GV) and second meiosis (MII) stage oocytes. **A** An outline of the experimental workflow for this study, in brief, using comparative transcriptome analysis and weighted gene co-expression network analysis (WGCNA) to characterise transcriptional signatures of bovine egg development. **B** The circular hierarchical clustering shows the differences between samples of different species; red means cattle, blue means sheep, green means pig and orange means mouse. **C**-**F** Hierarchical clustering heatmaps of GV and MII eggs for cattle, sheep, pigs and mice, respectively

In order to determine the specific vital genes that regulate the development of bovine oocytes from GV to MII stage, we carried out the following experimental design (Fig. 1A). Briefly, we conducted comparative transcriptomic analysis and WGCNA among the transcriptomic information of GV- and MII-stage oocytes from cattle, sheep, pigs and mice. Circular clustering dendrograms showed the transcriptional heterogeneity among species. Specifically, there were significant differences in transcriptome information among different species. In addition, there were differences in transcriptome data at GV and MII stages between species (Fig. 1B). Interestingly, the further individual clustering heat map of each species showed that the transcriptional heterogeneity of GV was lower than that of MII (Fig. 1C-F).

### Differential expression analysis

**Fig. 2:**
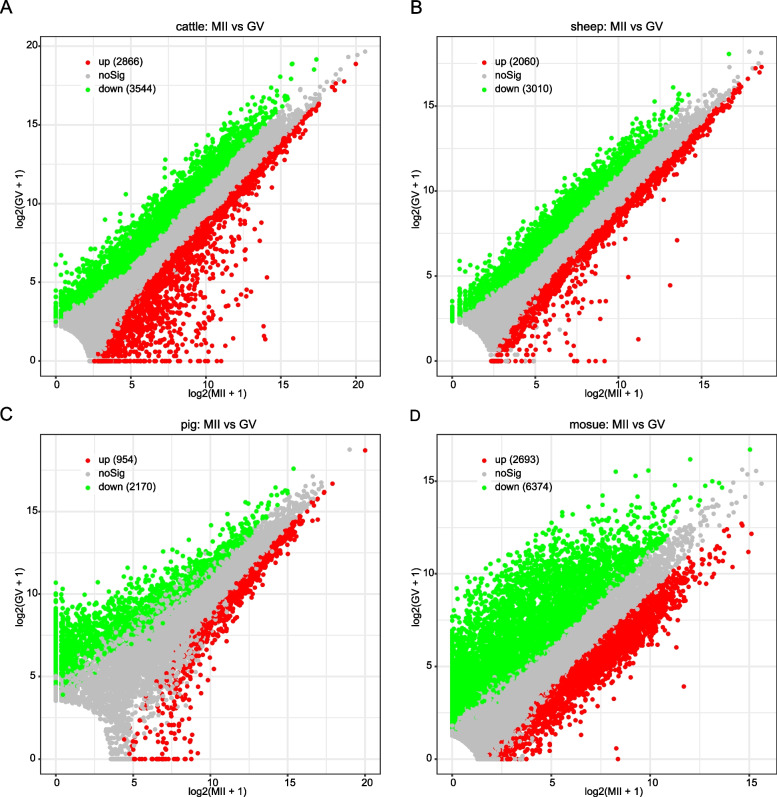
Differential analysis of oocyte development in GV and MII stages of different species. **A**-**D** Volcano plots sequentially show differentially expressed genes (DEGs) for oocyte development at GV and MII stages in cattle, sheep, pigs, and mice; red means up-regulated DEGs, green means down-regulated DEGs

To parse the transcriptional signatures from GV to MII stage in cattle, sheep, pigs and mouse oocytes, we performed differential expression analysis. A total of 6410 differentially expressed genes (DEGs) were obtained from bovine oocytes from GV to MII stage, of which 2866 genes were up-regulated and 3544 genes were down-regulated (Fig. 2A and Table S1). For sheep, 5070 DEGs were gained, including 2060 up-regulated and 3010 down-regulated genes (Fig. 2B and Table S2). A total of 3124 DEGs were identified in pig oocytes from GV to MII stage, of which 954 were up-regulated and 2170 were down-regulated (Fig. 2C and Table S3). The number of DEGs identified in the GV to MII stage of mouse oocytes was the largest, reaching 9067, of which as many as 6374 were down-regulated genes and only 2,693 up-regulated genes (Fig. 2D and Table S4).

### Transcriptional specificity in oocyte development from GV to MII stage in cattle compared to other species

**Fig. 3:**
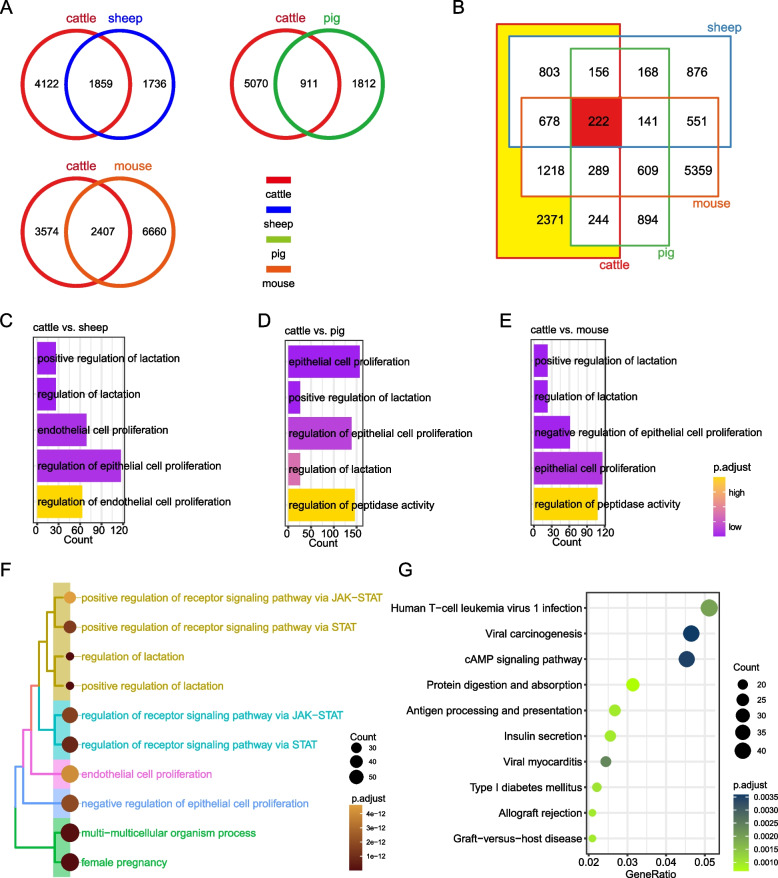
Comparative transcriptome analysis of oocyte development in cattle and other species. **A** Bicyclic Venn diagram showing transcriptional differences in cattle compared to other species alone during GV to MII. **B** Four-ring Venn diagram showing transcriptional differences in a comprehensive comparison of cattle and other species during GV to MII; red means cattle, blue means sheep, green means pig and orange means mouse. **C**-**E** Top 5 gene ontology (GO) terms of bovine-specific genes compared to sheep (**C**), pig (**D**) and mouse (**E**). **F** Top 10 GO terms of bovine-specific genes compared comprehensively with other species. **G** Top 10 Kyoto Encyclopedia of Genes and Genomes (KEGG) pathways of bovine-specific genes compared comprehensively with other species

We first determine the transcriptional similarities and differences in the development of oocytes from GV to MII in cattle *vs.* sheep, pigs and mice, respectively. The comparison between cattle and sheep showed that there were 1859 common genes between cattle and sheep, and 4122 genes were cattle-specific; For cattle and swine comparisons, there were 911 shared genes, and 5070 genes were cattle-specific; For bovine and mouse comparisons, there were 2407 genes in common and 3574 genes are bovine-specific (Fig. 3A). Further, we compared the transcriptional differences between cattle and sheep, pigs and mice as a whole, and found that 222 genes were conserved in the four species, while 2371 genes showed specificity in cattle (Fig. 3B). In order to explore the functions of bovine-specific genes, we performed functional enrichment analysis of the genes. We were surprised to find that ‘positive regulation of lactation’, ‘regulation of lactation’ and ‘endothelial cell proliferation’ were in the top 5 GO functional enrichment terms of bovine-specific genes regardless of whether cattle were compared with sheep, pigs or mice (Fig. 3C-E). In addition, ‘positive regulation of lactation’, ‘regulation of lactation’ and ‘endothelial cell proliferation’ also appeared in the top 10 GO terms of 2371 cattle-specific genes (Fig. 3F). Kyoto Encyclopedia of Genes and Genomes (KEGG) pathway analysis of 2371 cattle-specific genes suggested that the cAMP signalling pathway was significantly enriched (Fig. 3G).

### Functional analysis of genes regulating oocyte development from GV to MII stage in cattle and other species

**Fig. 4:**
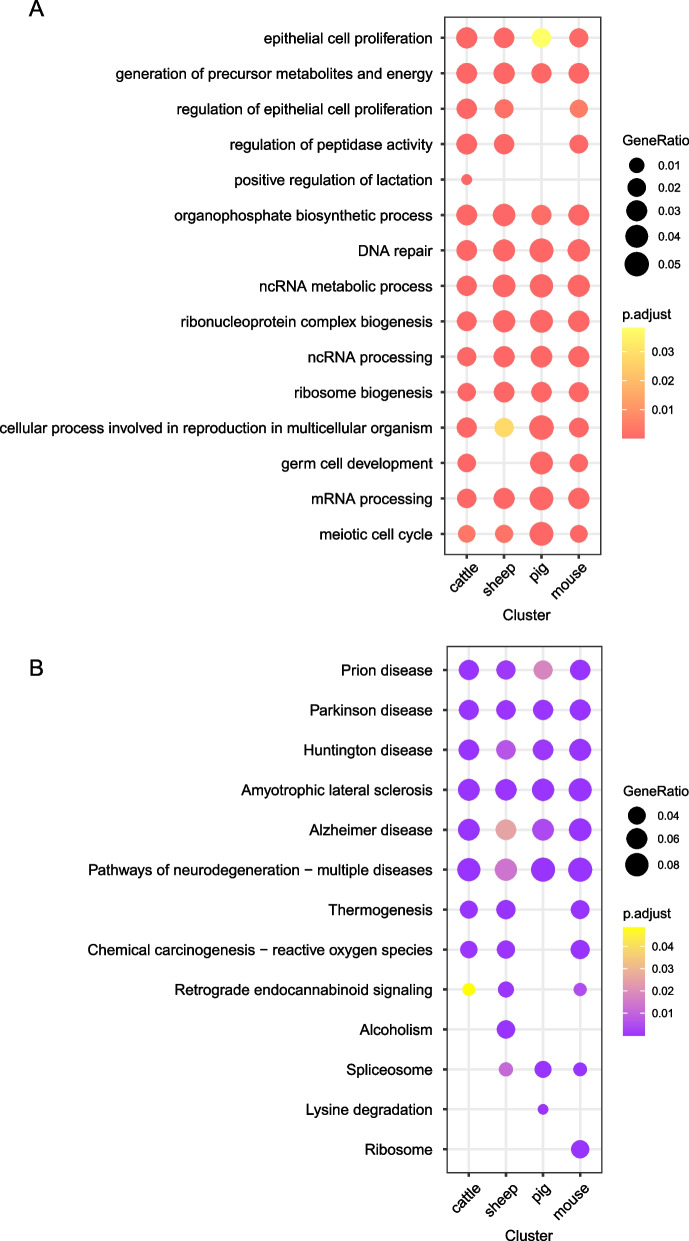
Integrated analysis of interspecies oocyte development during GV to MII stages. **A** Dot plot showing similarities and differences of the top 5 GO terms of all DEGs in four species, including cattle, sheep, pig and mouse. **B** Dot plot showing similarities and differences of the top 5 KEGG terms of all DEGs in four species

To gain a deeper understanding of the functions of DEGs during the development of cattle, sheep, pig and mouse oocytes from GV to MII stage, we performed a comparative analysis of DEGs in different species. For GO analysis, we found ‘positive regulation of lactation’ was enriched only in cattle; However, the processes of ‘generation of precursor metabolites and energy’, ‘DNA repair’, ‘ncRNA metabolic process’ and ‘ribonucleoprotein complex biogenesis’ were all enriched in the four species (Fig. 4A). In addition, no bovine-specific KEGG metabolic pathway was found to be enriched, and most of the pathways were enriched in the four species (Fig. 4B).

### WGCNA of oocyte development from GV to MII stage in multispecies

**Fig. 5:**
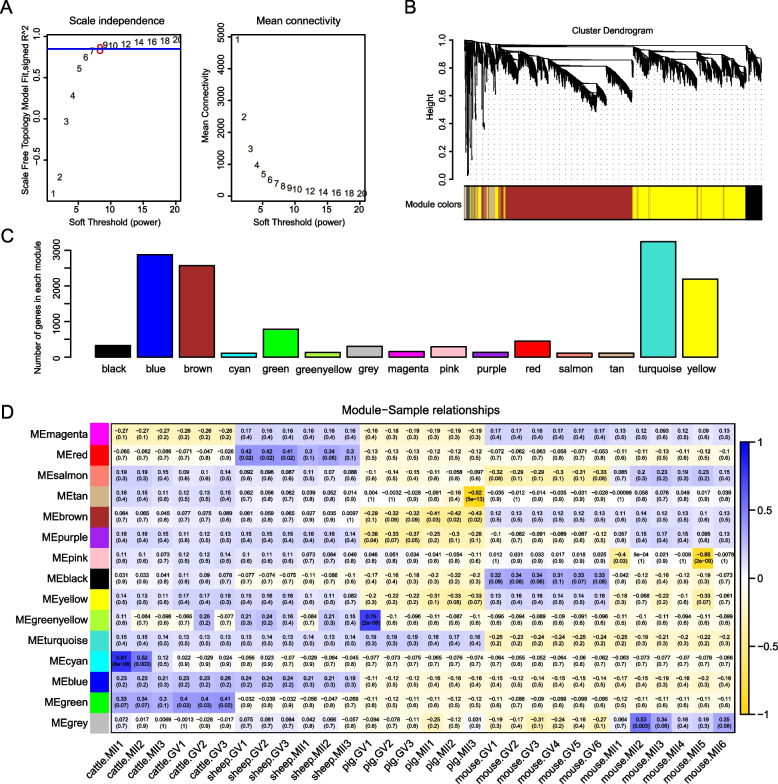
Weighted gene co-expression network analysis (WGCNA) of interspecies oocyte development during GV to MII stages. **A** Analysis of free-scale network topology for different soft-thresholding powers. **B** Hierarchical clustering plot showing the relationships of genes within modules. **C** Bar plot showing the number of gene in each module. **D** Heatmap showing the relationship between module and each sample; blue represents positive correlation, yellow represents negative correlation

To identify candidate genes that regulate the development of bovine oocytes from GV to MII stage, we employed WGCNA, an algorithm for constructing gene expression networks and phenotype relationships. To build a scale-free network, we choose a soft threshold = 8, with a scale-free topology fitting index *R*^2^ > 0.85 (Fig. 5A). Further, we used a one-step approach for module identification and merging (Fig. 5B). Fifteen modules were obtained, among which the cyan module had the least number of genes, only 107, and the turquoise had the most, reaching 3238 (Fig. 5C). The heatmap of module-sample relationships showed that the correlation between the module and a single sample was low, with only the correlation between the *cattle. MII1* sample and the cyan module being 0.81, and the rest of the relationships being < 0.8 (Fig. 5D).

### Identification of unique transcriptomic signatures for bovine oocyte development

**Fig. 6:**
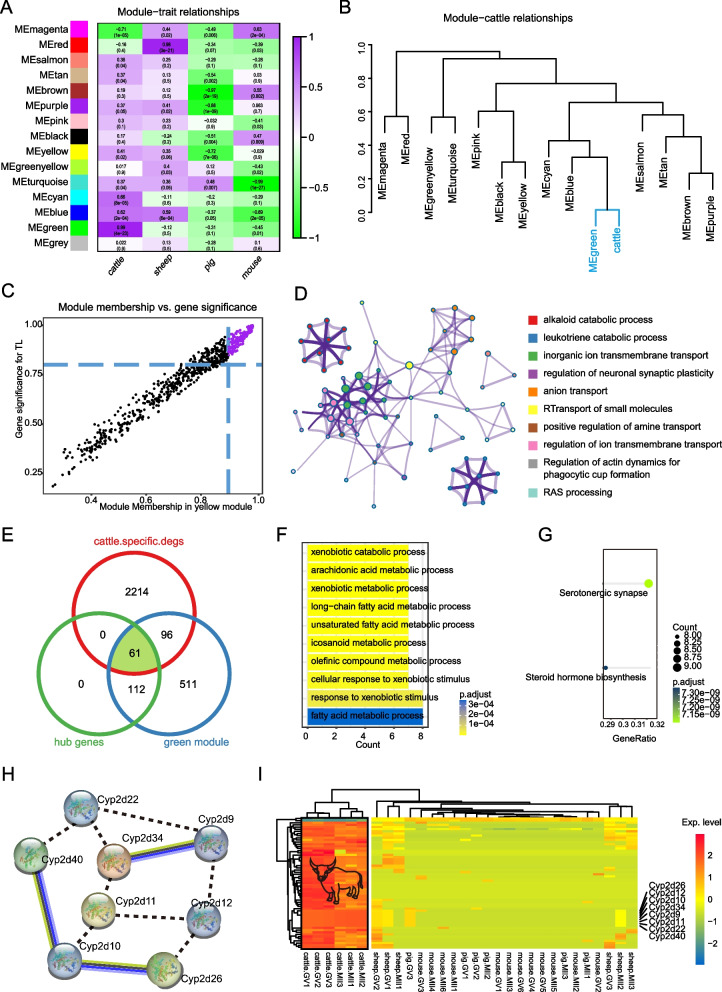
Identification of unique transcriptomic signatures in bovine oocyte development. **A** Heatmap showing the relationship between module and each trait; purple represents positive correlation, green represents negative correlation. **B** Hierarchical clustering plot showing the relationship between oocyte development of cattle and each module. **C** Scatter plot showing hub genes in green modules; purple dots represent hub genes and black dots represent non-hub genes within the green module. **D** Functional enrichment network of genes within green modules. **E** Three-ring Venn diagrams showing unique transcriptional signatures of bovine oocyte development. **F** Top 10 GO terms of 61 unique bovine transcriptional signature genes. **G** KEGG pathways of 61 unique bovine transcriptional signature genes. **H** The PPI network of unique bovine transcriptional signature genes. **I** Heatmap showing expression trends of 61 bovine-specific trait genes across multiple species

In order to identify the specific regulatory gene signatures of bovine egg development, we performed a correlation analysis between species information and the gene scale-free expression network, and the results showed that the green module was closely related to bovine oocyte development, correlation = 0.99, *p* = 4e − 23 (Fig. 6A). Next, we combined bovine species information and module information into a matrix for correlation analysis, and hierarchical clustering suggested that the green module was related to the regulation of bovine oocyte development (Fig. 6B). Once we determined that the green module was related to the specific regulation of bovine oocyte development, we tried to find the hub genes within this module, and the results showed that a total of 173 hub genes were identified (Fig. 6C). The functional enrichment analysis network found that these hub genes were involved in the regulation of the RAS signalling pathway (Fig. 6D). We comparatively analysed cattle-specific DEGs, green module genes and hub genes, and finally obtained 61 unique transcriptomic signatures of bovine oocyte development (Fig. 6E, Table S5). The top 10 GO terms showed that unique transcriptomic signatures were heavily involved in substance metabolism, such as fatty acid metabolic processes (Fig. 6F). KEGG pathway analysis showed they participated in regulating steroid hormone biosynthesis (Fig. 6G). The Protein–protein interactions (PPI) analysis indicated that there was a protein interaction network for CYP2d family proteins (Fig. 6H). In cattle, sheep, pigs and mice, these genes showed transcriptional signatures that were highly expressed in cattle (Fig. 6I).

## Discussion

Assisted technology is increasingly used to achieve pregnancy in the production of important agricultural animals [16]. However, most artificial reproduction methods have achived limited success, including in vitro embryo production. While many factors can lead to lower pregnancy rates relative to natural reproduction, female egg development abilityis one of many limiting factors. Screening of specific candidate genes and identification of unique transcriptomic signatures by means of multi-species comparisons are necessary to improve insights into bovine oocyte development. In this study, we assessed the transcriptional signatures of bovine, ovine, porcine and mouse eggs developing from the GV to MII stage. The specific genes of bovine compared to other species were also screened to provide a theoretical basis for further understanding of bovine oocyte development.

Differential expression analysis suggested that most genes were down-regulated as oocytes progressed, which was consistent with previous studies [17], as the transcriptional activity of mammalian oocytes was silenced during the GV period before the meiotic restart [18, 19]. After transcriptional silencing, the oocyte genome undergoes the first and second meiosis, and the transcriptional activity is not restored until cleavage of the early embryo after fertilisation [20]. It is worth mentioning that during male spermatogenesis, since transcriptional silencing does not occur, our previous study found that most genes were up-regulated before and after sperm maturation, which promoted sperm maturation [15].

Once we obtained DEGs from different species, we attempted to compare the differences between bovine and other species to gain a deeper insight into bovine oocyte development. Comparative analysis found that bovine-specific DEGs were involved in the ‘female pregnancy’ when comparing cattle with sheep, pigs or mice. As expected, the ‘cAMP signalling pathway’ was also enriched. A high expression level of cAMP plays a vital role in maining the meiotic arrest [21, 22]. Except for pigs, the oestrus cycle of cattle is significantly longer compared with other species [23]. Secondly, compared with the other three species, cattle have a longer gestation period, reaching 283 days [24], which may be one of the reasons why bovine oocytes reappear more cAMP signalling-regulated genes during development. Moreover, comparative analysis of gene set functions found that ‘DNA repair’ occurred during oocyte development in all four species. A recent and interesting study suggested that there may be species differences in the ability of GV and MII stage oocytes to perform DNA repair [25].

WGCNA has been widely used to identify and characterise key pathways and genes [15, 26]. Under the conditions of building a scale-free gene topology network, we obtained a total of 15 gene function modules, where the grey module refers to the gene set that has not yet been aggregated in any module. We identified green modules as closely associated with bovine oocyte development (correlation = 0.99, *p* = 4e − 23). Next, 173 hub genes involved in the ‘RAS signalling pathway’ were obtained. The RAS signalling pathway is involved in the transmission of intracellular signals and plays a role in cell growth, differentiation and survival [27, 28]. A study completed by Gibbs reported that RAS was able to induce GVBD in oocytes [29]. The unique transcriptional signatures described above are all closely related to the developmental process of bovine oocytes. Further, we integrated comparative transcriptome analysis and WGCNA to identify 61 bovine-specific signature genes (Fig. 6). Unexpectedly, these signature genes were mainly involved in metabolic regulation and steroid hormone biosynthesis, and some cytochrome P450 (CYP) family members were found. More interestingly, we found that 61 signature genes, including members of the CYP family, had highly expressed forms in cattle, compared with other species.

## Conclusion

In conclusion, this study identified 61 bovine-specific signature genes by integrating the comparative transcriptome and WGCNA, revealing the unique transcriptional signature of bovine compared to other species. This study provides a theoretical basis for elucidating the unique developmental regulation of bovine oocytes. However, more experimental studies are needed to mine and identify targets that regulate bovine oocyte development.

## Materials and methods

### RNA-seq data acquisition

All gene expression data (RNA-seq) concerning oocyte development from germinal vesicle (GV) to metaphase II (MII) were downloaded from the public database, including the gene expression omnibus (GEO) and genome sequence archive (GSA) database. Specifically, these included cattle [GAS database, CRA005589] [30], pig [GEO database, GSE160334] [9], sheep [GEO database, GSE148022] [8], and mouse [GEO database, GSE119906].

### Workflow for raw RNA-seq data

In order to avoid errors introduced by different bioinformatics analysis methods in different studies, we used standardised and strict processes to process the raw RNA seq data of different species. Briefly, FastQC (v0.11.8) and Fastp (v0.23.1) were used to process sequencing data adapters and filter low quality bases and reads [31]. Next, STAR (v2.7.0f) was used to map the reads to reference genome, and generate a BAM format file by using the ‘*–outSAMtype BAM SortedByCoordinate*’ parameter [32]. The reference genome version of *Bos taurus* was ARS-UCD1.2, *Ovis aries* was ARS-UI_Ramb_v2.0, *Sus scrofa* was Sscrofa11.1 and *Mus musculus* was GRCm38.p5. Finally, a gene expression matrix was generated by FeatureCounts (v1.6.3) [33].

### Hierarchical cluster analysis

Hierarchical cluster analysis was used to observe the between-group variability of samples. The normalised fragments per kilobase of exon model per million mapped fragments (FPKM) matrix as input and use the *hclust* function for hierarchical cluster analysis. Heatmaps (pheatmap, R package, v1.0.12) and ring treemaps (ggtree, R package, v3.2.1) were used to visualise the results [34].

### Identification of differentially expressed genes (DEGs)

The DESeq2 (R package, v1.32.0) was used to find the DEGs and the threshold of DEGs was ‘|log2fold change|> 1 and *p*-value < 0.05’ [35]. Briefly, the raw reads count was used as the input of DESeq2, using the *DESeqDataSet* function for data preprocessing, and the *DESeq* function for differential expression analysis. The principle of *DESeq* was empirical Bayes shrinkage for dispersion estimation [35].

### Multispecies comparative transcriptomic analysis of oocyte development

To gain deeper unique transcriptomic signatures of bovine oocyte development, we compared the transcriptome of cattle and other species. Briefly, we converted gene IDs of different species into a uniform gene symbol by using gprofiler2 (R package, v0.2.1) [36]. Due to differences in annotation levels between species, we only compared genes capable of homologous switching in different species. Next, we compared the DEGs of other species with that of bovines, respectively. Venn diagrams were used to demonstrate the peculiarities of bovine oocyte development.

### Weighted gene co-expression network analysis (WGCNA)

To uncover the correlation between genes and bovine oocyte development, the WGCNA (R package, v1.70–3) was selected to perform WGCNA [37]. In short, the gene expression matrices for all species were *log2 (x* + *1)* normalised. Next, to ensure that a scale-free network is constructed, the R^2^ of the soft threshold was set to a minimum of > 0.85. It is worth noting that we used a one-step approach for module screening, and the primary modules were filtered and merged according to the default values. Bar plots, heatmaps, and dendrograms were used to visualise the results.

### Identification of hub genes

Hub genes refer to the genes with high connectivity in a module identified by WGCNA, which are often closely related to traits. First, we selected a candidate module related to bovine oocyte development according to correlation analysis heatmaps and hierarchical clustering trees. Next, the judgement standard of the hub gene was module membership (MM) > 0.9 and gene significance (GS) > 0.8 [26].

### Function enrichment analysis

DEGs were processed for gene ontology (GO) and the Kyoto Encyclopedia of Genes and Genomes (KEGG) pathway analysis by using clusterProfiler (v4.0.5) [38]. We used the gene sets converted to mouse IDs as input to clusterProfiler and used the *org.Mm.eg.db* annotation collection mouse KEGG annotation library for functional annotation [39–41]. Moreover, due to the limited number of gene sets and database complexity, the function enrichment network was performed using Metascape (http://metascape.org/gp/index.html).

### Acquisition of signature genes and protein–protein interaction (PPI) network analysis

A signature gene means that it is both a hub gene and a DEGs. Signature genes were performed the PPI network analysis through STRING (v11.5) (https://string-db.org/) to observe the interaction between genes.

## Supplementary Information

**Additional file 1.**

## Data Availability

The datasets analyzed during the current study are available in public database; Cattle [GAS database, CRA005589, https://ngdc.cncb.ac.cn/gsa/browse/CRA005589], pig [GEO database, GSE160334, https://www.ncbi.nlm.nih.gov/geo/query/acc.cgi?acc=GSE160334], sheep [GEO database, GSE148022, https://www.ncbi.nlm.nih.gov/geo/query/acc.cgi?acc=GSE148022], and mouse [GEO database, GSE119906, https://www.ncbi.nlm.nih.gov/geo/query/acc.cgi?acc=GSE119906].

## Abbreviations

LH: Luteinising hormone
GV: Germinal follicle
MII: The second meiosis
cAMP: Cyclic AMP
PKA: Protein kinase A
MPF: Meiosis-promoting factor
GVBD: GV break down
RNA-seq: RNA sequencing
GEO: Gene expression omnibus
GSA: Genome sequence archive
PPI: Protein–protein interaction
WGCNA: Weighted gene co-expression network analysis
DEGs: Differentially expressed gene
GO: Gene ontology
KEGG: Kyoto Encyclopedia of Genes and Genomes
CYP: Cytochrome P450
FPKM: Fragments per kilobase of exon model per million mapped fragments
